# Amplification of *P. falciparum* Cytoadherence through Induction of a Pro-Adhesive State in Host Endothelium

**DOI:** 10.1371/journal.pone.0024784

**Published:** 2011-10-17

**Authors:** Yang Wu, Tadge Szestak, Monique Stins, Alister G. Craig

**Affiliations:** 1 Liverpool School of Tropical Medicine, Liverpool, United Kingdom; 2 RT Johnson Division of NeuroImmunology, Johns Hopkins School of Medicine, Baltimore, Maryland, United States of America; Université Pierre et Marie Curie, France

## Abstract

This study examined the ability of *P.falciparum*-infected erythrocytes (IE) to induce a pro-adhesive environment in the host endothelium during malaria infection, prior to the systemic cytokine activation seen in the later phase of disease. Previous work had shown increases in receptor levels but had not measured to actual impact on IE binding. Using a co-culture system with a range of endothelial cells (EC) and IE with different cytoadherent properties, we have characterised the specific expression of adhesion receptors and subsequent IE binding by FACS and adhesion assays. We have also examined the specific signalling pathways induced during co-culture that are potentially involved in the induction of receptor expression. The results confirmed that ICAM-1 is up-regulated, albeit at much lower levels than seen with TNF activation, in response to co-culture with infected erythrocytes in all three tissue endothelial cell types tested but that up-regulation of VCAM-1 is tissue-dependent. This small increase in the levels of EC receptors correlated with large changes in IE adhesion ability. Co-culture with either RBC or IE increased the potential of subsequent adhesion indicating priming/modulation effects on EC which make them more susceptible to adhesion and thereby the recruitment of IE. Trypsin surface digestion of IE and the use of a *Pfsbp1*-knockout (ko) parasite line abrogated the up-regulation of ICAM-1 and reduced IE binding to EC suggesting that PfEMP-1 and other molecules exported to the IE surface via the PfSBP1 pathway are major mediators of this phenotype. This was also supported by the higher induction of EC adhesion receptors by adherent IE compared to isogenic, non-adherent lines.

## Introduction

*Plasmodium falciparum* infection is a major cause of severe disease associated with a range of clinical syndromes including cerebral malaria (CM). The exact nature of the pathology underlying severe malaria is not fully understood but two main mechanisms have been considered, namely cytoadherence and inflammation. Organ specific pathogenesis such as that seen in CM is believed to be mediated by sequestering infected erythrocytes (IE) on endothelial cells (EC), and/or by rosetting between infected and uninfected erythrocytes to form clumps in the microvasculature of major organs [1]–[4]. Several immune and inflammatory mediators are likely to be heavily implicated in the process leading to this [5], [6]. Factors such as high levels of plasma pro-inflammatory cytokines and circulating immune-complexes might play a role in activating/damaging EC in the course of infection [7].

The primary mechanism of cytoadherence of the asexual-stage *P. falciparum* IE is complex involving a range of host receptors (e.g. intercellular adhesion molecule-1 (ICAM-1), CD36, CD31 and P-selectin) interacting with a family of parasite-encoded proteins (mainly the variable and diverse *var* gene products, encoding PfEMP-1 (*Plasmodium falciparum* erythrocyte membrane protein-1)), that are displayed on the surface of IE [8]. This is further complicated by the ability of these receptors to cooperate in achieving efficient IE binding to EC [9]–[11]. Additionally, the production of high levels of TNF and other pro-inflammatory mediators is a key feature of malaria infection which contributes to the systemic and organ-related malaria syndromes [12]. These pro-inflammatory cytokines can produce endothelial activation by up-regulating or *de novo* synthesis of adhesion receptors and cytokines to increase sequestration of PRBCs within the microvasculature and, in some cases, contribute to the development of chronic inflammation by recruiting leukocytes into tissues [13], [14]. For example, ICAM-1 is markedly up-regulated in severe malaria and has been implicated as being involved in progression to cerebral disease [15]–[17].

Correlation between disease and cytoadherence phenotype is not simple but has been demonstrated in a number of cases [3], [18], [19]. Using *var* gene upstream regions as molecular tags, additional associations between PfEMP1 variant type and severe malaria have been revealed [20], [21]. Many studies have suggested that adhesion molecules play a role in malaria pathogenesis and patient survival [22], [23]. However, in the early stages of infection although some indication of inflammation has been noted in human experimental infections [24], elevated adhesion molecule expression such as ICAM-1 has not been observed. Thus the parasite faces a challenge to modify the host environment to support IE adhesion during the early stages of infection in the erythrocytic cycle; the ability to modulate the host environment to produce efficient cytoadherence would be of great benefit to parasite survival and transmission.

A number of groups have examined the effect of IE on EC responses in co-culture systems with respect to functional outcomes on EC such as cell adhesion, cell apoptosis and/or survival, inflammatory responses and signal transduction. The first report was delivered by Udeinya and Akogyeram in 1993, who showed induction of adhesiveness in HUVEC by *P.falciparum*-infected erythrocytes up to 250% and that this induction was related to direct contact via EC adhesive ligands [25], [26]. In 2005, Viebig *et al* showed direct induction of ICAM-1 on HUVEC by IE [27], while other work by our group showed the induction of ICAM-1 required low concentrations of TNF [28]. A fourth study has demonstrated that IE alone do not have the ability to induce ICAM-1 in HUVEC but could do so in brain-derived EC [29]. In these experiments, IE induced dose- and exposure-dependent ICAM-1 up-regulation in HBMEC was seen, with both membrane-associated IE and parasite-derived soluble factors involved in this process. Co-culture of IE also induced nuclear translocation of NF-κB in HBMEC, which is linked to the ICAM-1 expression [7], [29]. All of these studies have confirmed that cell-cell contact is a critical step for the activation of endothelial cells and the required direct physical contact can be as little as 30 to 60 minutes [29]. In all cases there was no requirement of high doses of TNF for adhesion molecule induction. These responses would be expected to produce a pro-adhesive state increasing subsequent IE cytoadherence, as also demonstrated for IE-induced ectophosphorylation of CD36 [30], but this has not been performed. Therefore there is a need to extend this work using multiple endothelial cell types and parasite lines to verify the hypothesis that increased cytoadherence can be mediated by previous IE exposure and to understand the mechanisms underpinning this.

Most investigations studying the role of IE on endothelial activation use HUVEC [31] and human dermal microvascular endothelial cells (HDMEC) [32], both of which are primary cells and approximate the physiological features of *in vivo* cells more closely than cloned or transformed cell lines but are not derived from one of the key tissues involved in cerebral malaria, namely the brain. There are some examples of the use of primary brain-derived EC, mainly from the Stins group [33]. These cells, which possess the ability to form tight junctions, are critical to the integrity of the blood-brain barrier and serve as the best *in vitro* model for understanding the impact of parasite-EC interactions in cerebral malaria (CM) [34], [35]. However they are difficult to obtain and are routinely replaced by immortalised lines that resemble the behaviour of primary cells. This is important as different EC subtypes vary widely in their expression profiles; for example, HUVEC do not express CD36 nor the chemokine receptor CXCR4 [36]; HDMEC express CD36; human brain microvascular endothelial cells (HBMEC) express CXCR4 [28]. ItG, A4 and C24 [37], [38] are isogenic parasite lines that have distinctive cytoadherence properties; ItG binds strongly to ICAM-1 and to CD36, A4 binds strongly to CD36 and to ICAM-1 moderately, and C24 binds strongly to CD36 but binding to ICAM-1 is minimal. These strain-specific properties, based on variable expression of PfEMP1, enable different IE-EC interactions [39]. In this study we have used an *in vitro* co-culture model where intact IE are placed in direct contact with different human EC monolayers (different in the adhesion receptors displayed and their responsiveness to inflammatory cytokines) to evaluate whether co-culture with normal or parasitized erythrocytes (with adhesive and non-adhesive lines) activate endothelial cells *in vitro* and particularly whether they up-regulate adhesion molecule expression that translates into a pro-adhesive effect.

## Results

### Expression levels of endothelial cell adhesion molecules

**Figure 1:**
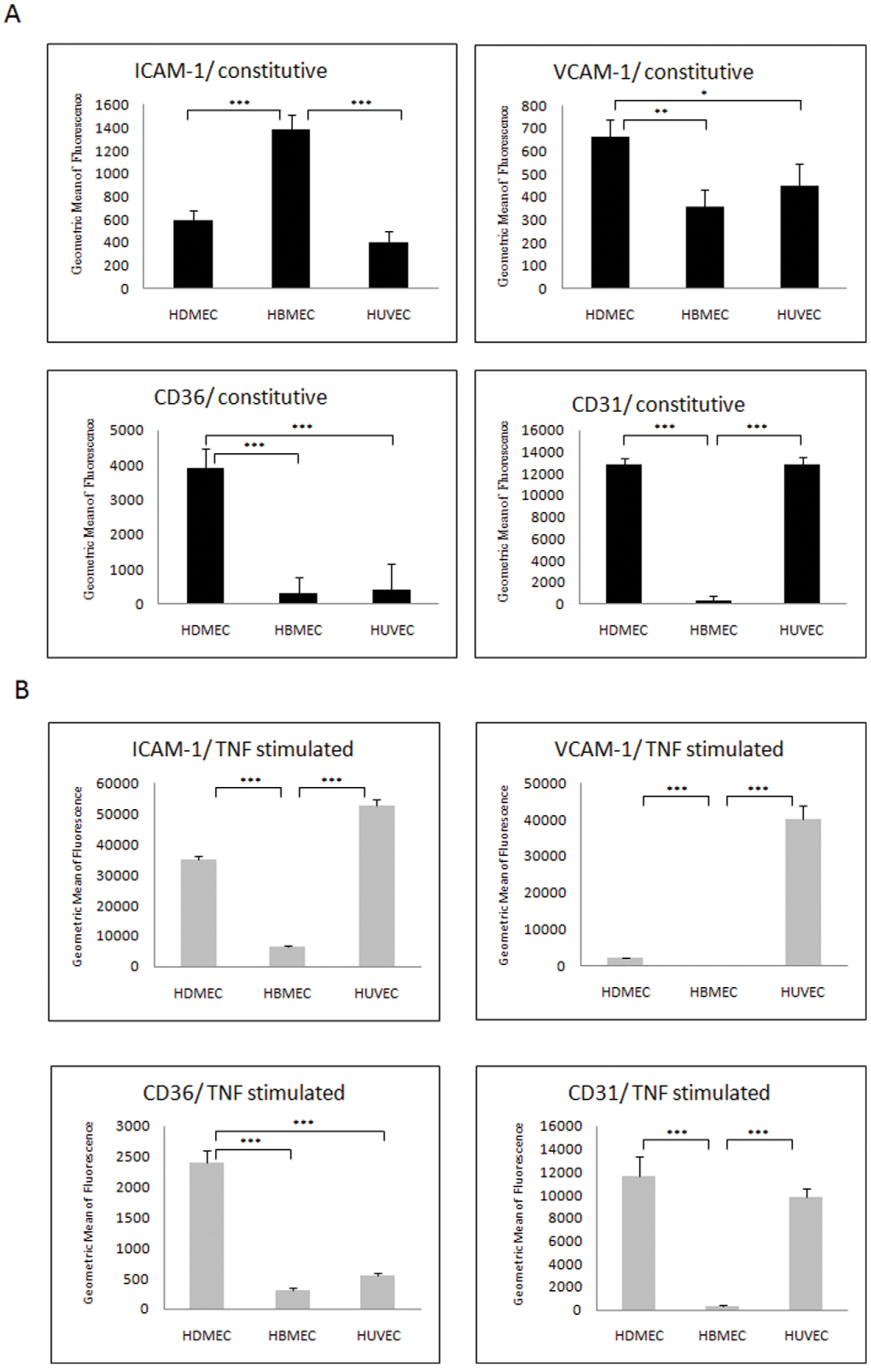
Constitutive and induced levels of endothelial receptor expression. (A) Basal expression levels of four adhesion receptors in three EC lines. Healthy confluent ECs were detached by accutase and labelled with antibodies. The expression levels were determined by FACS. (B) Confluent EC were stimulated with TNF at 1 ng/ml for overnight (16 hours), the cells were detached and the expression levels of four adhesion receptors were determined by FACS. The expression level was represented as the geometric means of fluorescence intensity from three independent experiments. Data were analysed by comparing means of each group from three independent experiments, means ± SD are presented. *P<0.05; **P<0.001; ***P<0.0001.

Adhesion molecules were expressed at different levels in the three un-stimulated endothelial cell lines. As seen in Figure 1A, HBMEC had the highest constitutive expression level of ICAM-1, significantly higher than HDMEC and HUVEC (p<0.001). HDMEC had the highest constitutive expression level of CD36, significantly higher than HBMEC and HUVEC (p<0.001). The constitutive expression level of CD31 in HBMEC was significantly lower than both HDMEC and HUVEC (p<0.001). The levels of VCAM-1 were slightly higher in HDMEC compared to HBMEC and HUVEC. In response to TNF stimulation all three cell types increased ICAM-1 expression significantly, although HUVEC had the highest levels while HBMEC had relatively much lower expression levels. VCAM-1 expression was significantly increased only in HUVEC. CD36 was significantly decreased after TNF stimulation in HDMEC but was still relatively high compared to the levels seen for HUVEC and HBMEC, which remained unaffected (and at low levels) by TNF. CD31 levels showed, as expected, no significant changes on FACS after TNF stimulation on all three EC types (Figure 1B).

### Kinetics of ICAM-1expression in the three endothelial cell lines

We used ICAM-1 as an example to measure the dynamic changes in receptor expression after co-culture with IE (ItG in this experiment) with the three endothelial cell types. Co-culture of ItG with HBMEC or HUVEC induced a significant increase in ICAM-1 expression starting from 3 hours co-culture, while in HDMEC it took 8 hours to produce a significant increase. However, the maximum ICAM-1 induction in all three endothelial cell lines was overnight co-culture (16 hours). The rate of increase in HBMEC was greater than that in HDMEC and HUVEC (data not shown).

### Expression patterns of EC adhesion molecules in co-culture models

**Figure 2:**
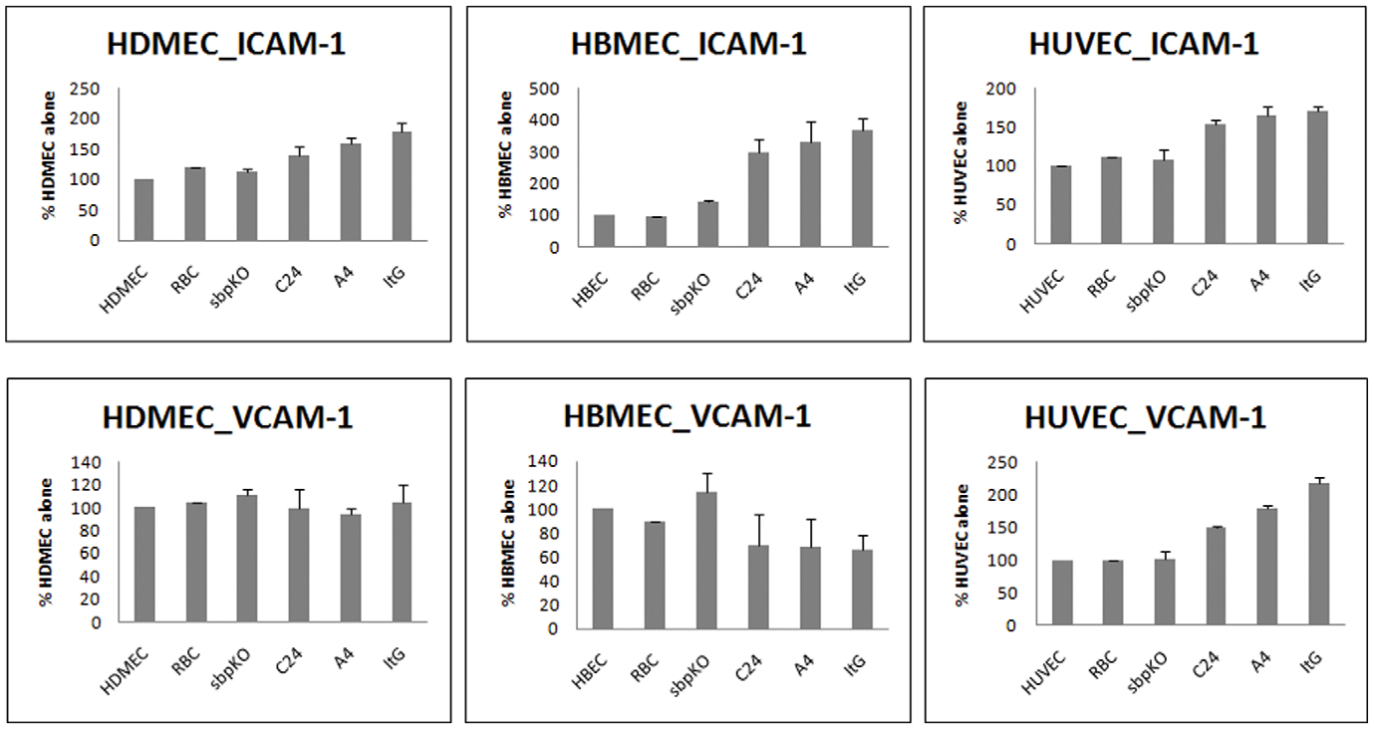
Expression of EC adhesion receptors after overnight co-culture with parasite lines: *Pfsbp* knockout, C24, A4 and ItG and normal RBC. The adhesion molecules on three EC types were measured by FACS. Data were presented as the mean percentage of response induced by EC alone ± S.D (n = 3). The increase in ICAM-1 levels in three EC types after co-cultured with parasite lines C24, A4 and ItG were extremely significant compared to co-culture with no-parasite or normal RBC (p<0.0001). The increase is also very significant compared with the level induced by the *Pfsbp* knockout *line* (p<0.001). The increase in VCAM-1 after co-culture with C24, A4 and ItG lines compared to no-parasite, RBC and *Pfsbp* knockout line was extremely significant in HUVEC only (p<0.0001).

Variable levels of receptor expression on EC in response to IE co-culture were observed (Figure 2 and Figure S1).

#### ICAM-1

The expression of ICAM-1 increased significantly compared to EC alone after co-culturing with IE for the three parasite lines with all three endothelial cell types regardless of ICAM-1-binding (A4 and ItG) or non-binding (C24) parasite strains, with a gradation in response such that ItG>A4>C24. However the levels of ICAM-1 in all three EC lines on co-culture were significantly higher than co-culture with the *Pfsbp1* knockout parasite line only when the ICAM-1-binding lines A4 and ItG were used.

#### VCAM-1

The expression of VCAM-1 increased significantly after co-culture with IE for all three parasite lines in HUVEC only and these effects were significantly higher than co-culturing with the *Pfsbp1* knockout strain. There was no significant induction or inhibition of VCAM-1 in HDMEC or HBMEC.

#### CD31

Co-culture with IE with all three PfEMP1-expressing parasite lines decreased CD31 expression in the three EC types, although the percentage changes were different. There appeared to be a small but consistent reduction in CD31 levels on HUVEC and HDMEC when cultured with IE (regardless of the expression of PfEMP1). The consistent reduction seen with HBMEC is interesting but it should be noted that the constitutive expression level of CD31 in this EC line was very low, so percentage changes may be misleading.

#### CD36

All three EC types responded on IE co-culture with a trend towards decreased CD36 levels compared to EC alone. However the constitutive expression levels of CD36 in HUVEC and HBMEC were very low, about 10 fold lower than HDMEC, therefore, the trend for a decrease in expression in these cell types needs to be considered carefully. The reduced level of expression seen in comparing EC alone and co-culture with ItG in HDMEC was statistically significant (p<0.01) but did not translate into changes in C24 adhesion (see below).

### Previous IE-EC exposure increases subsequent IE binding

**Figure 3:**
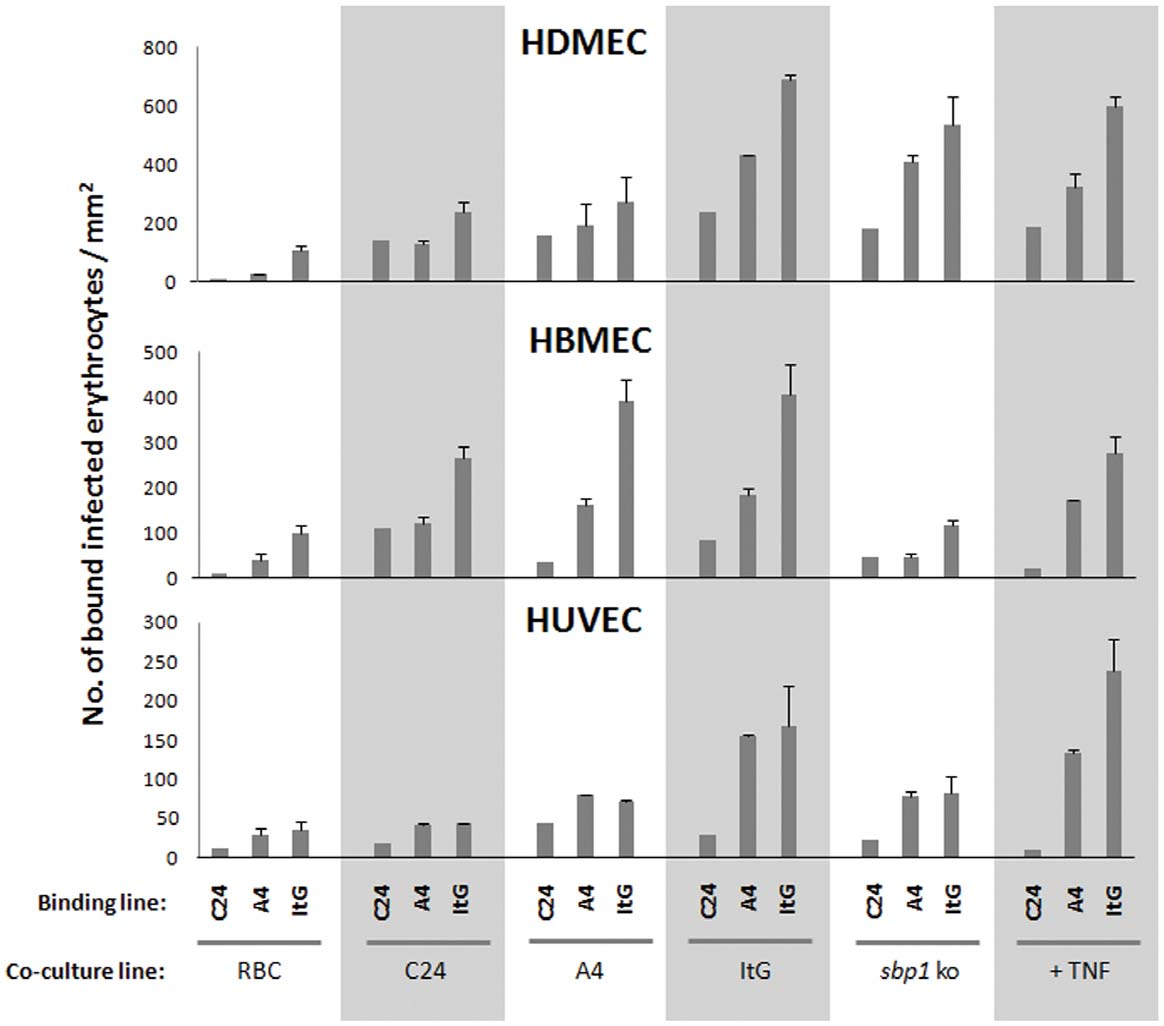
Changes in static binding to EC modulated by co-culture with RBC, non-adhesive and adhesive parasite lines. Confluent EC were co-cultured with RBC or IE for 16 hours, then the overlay cells were washed away and subsequently the EC were analysed for their adhesion ability by static binding assays as described in methods section. Data shown (see also Table S1) are the mean number of IE per mm^2^ ± S.E of the means of at least 4 independent experiments.

The previous section indicated increased levels of IE receptor expression on co-culture but we wanted to understand the impact of this on subsequent IE binding ability. The three endothelial cell types exhibited different adhesion patterns after co-culture with the parasite variants. Figure 3 shows the IE binding results following overnight co-culture with different parasite variants and controls.

These data offer a complex picture of IE binding after co-culture with an indication of at least two types of EC modulation taking place, one dependent on a binding interaction via PfEMP1 and the other based on contact with IE. To investigate this we have carried out a set of comparisons to address the questions listed in Table 1, which also shows the p-values of these comparisons:

1. In general, co-culture with IE induced higher levels of cytoadherence than the very low levels seen after RBC co-culture (Question 1) or basal levels of adhesion (Figure S3). This was only statistically significant in HBMEC and HUVEC, probably due to the constitutive CD36-dependent adhesion seen with HDMEC.
2. Parasite lines with strong binding to EC (e.g. ItG) produced higher subsequent cytoadherence than non/lower binding lines (e.g. C24) in HDMEC, but this was not statistically significant with HBMEC and HUVEC (Question 2).
3. The loss of PfSBP1 abolishes PfEMP-1 trafficking to the erythrocyte membrane surface, such that PfEMP-1 is not displayed and resulting in loss of adherence [40], [41]. Induction of pro-adhesiveness was higher in HBMEC co-culture with the non-transgenic parasite lines (C24, A4 and ItG) compared to *Pfsbp1*ko (Question 3), although the effects in HUVEC and HDMEC were not significant. For HDMEC, which showed roughly equivalent levels of induced adhesion with ItG and *Pfsbp1*ko co-culture, this may reflect the high constitutive levels of CD36 available on this EC type. The lack of statistical significance with HUVEC may reflect the large variability seen in the binding assays for ItG co-culture/ItG binding seen with this EC (Table S1).
4. In HDMEC and HUVEC binding assays, the level of adhesion induced by co-culture with the *Pfsbp-1* knockout line was higher than co-culture with RBC (Question 4).

**Table 1:** Statistical comparisons (two-tailed t-test).

		p-value		
Question	Comparison	HDMEC	HBMEC	HUVEC
1	RBC/ItG binding vs A4/ItG binding	0.1200	0.0029	0.0007
2	C24/ItG binding vs ItG/ItG binding	0.0004	0.1652	0.1185
3	sbp1 ko/ItG binding vs ItG/ItG binding	0.2072	0.0253	0.4092
4	sbp1 ko/ItG binding vs RBC/ItG binding	0.0030	0.3125	0.0001

1. Does co-culture with IE lead to induction of higher levels of binding compared to RBC co-culture?

2. Are higher levels of binding induced in all three EC by co-culture with ItG (ICAM-1-binding) compared to co-culture with C24 (non ICAM-1-binding)?

3. Do adherent IE (e.g. ItG) induce higher levels of binding on co-culture than the sbp1 ko parasite line?

4. Can co-culture with the sbp1 ko parasite line also induce increased levels of cytoadherence?

### Trypsin treatment of IE abrogates the up-regulation of ICAM-1 on HUVEC caused by co-culture with the parasite line ItG

**Figure 4:**
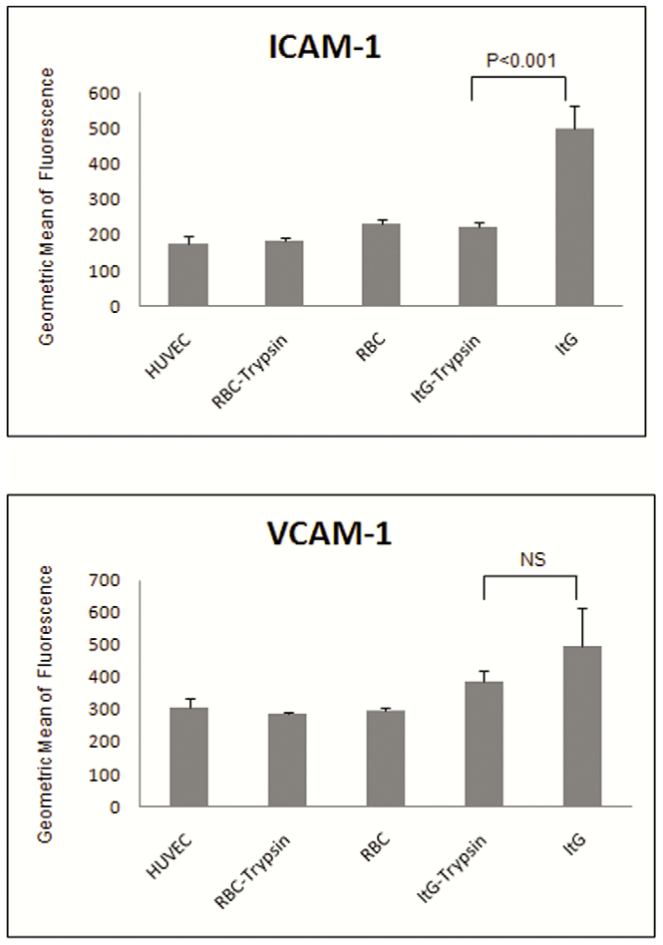
Trypsin treatment modulates the up-regulation of ICAM-1 on HUVEC caused by co-culture with the adhesive parasite line ItG. Trophozoite stage ItG IE were treated with 0.1 mg/ml trypsin or mock buffer for 12 min, then were used in co-culture for 16 hours with HUVEC. ICAM-1 expression on the endothelial cells was measured by FACS and represented as the geometric means of fluorescence intensity. Data shown are the means ± S.D. Reduced up-regulation for both molecules of 56% for ICAM-1 (p<0.001) and 22% for VCAM-1 (n.s.) was observed.

As seen in Figure 2, co-culture with IE, particularly adhesive parasite lines, produces a significant induction of adhesion molecules, including ICAM-1. This effect has been shown previously to require direct cell-cell contact [29], [30] but not necessarily adhesion [29]. In this experiment (Figure 4) we found that co-culture of ItG and HUVEC induced significant up-regulation of ICAM-1 but that trypsin treatment of co-cultured IE, which removes PfEMP1 (and other proteins) from the IE surface, reduced up-regulation of ICAM-1 (p<0.01), in a similar manner to that seen with co-culture with the *Pfsbp1* ko line and confirming our earlier findings.

### Cell signaling pathways induced in endothelium by infected erythrocyte co-culture

**Figure 5:**
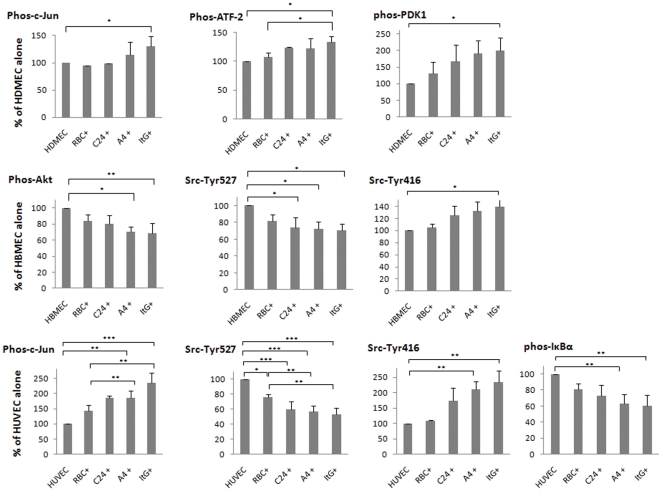
Signalling pathways showing significant changes due to co-culture with IE of parasite lines with different adhesive properties. Endothelial cells were co-cultured with IE for 20 min, then the activation of signalling components were measured by Western blot and quantitated by intensity scan. Statistical analysis was carried out on three independent experiments. The results are represented as a ratio to the constitutive signalling level seen in the corresponding EC alone sample. Data shown are the means of % response compared to control (EC alone) ± S.D. *P<0.05; **P<0.001; ***P<0.0001.

To understand the signalling pathways involved in both ICAM-1-dependent and ICAM-1-independent modulation of EC, five signalling pathways involving 10 signalling transduction molecules (Table 2) were measured upon co-culture of different IEs with the three EC types. Shown in Figure 5 are the pathways showing significant changes after 20 min co-culture with RBC or IE. In HBMEC the Src pathway was activated on contact with IE, although only co-culture with ItG reached statistical significance. In HDMEC, MAPK (c-Jun) and PI3 (Akt) pathways were both activated. In HUVEC, MAPK (c-Jun) and Src pathways were activated, and the NF-κB pathway was also activated as shown by the reduction of total IκBα protein in the cytosol after co-culture with adhesive parasite lines (A4 and ItG). This suggested cytoadherence dependent signalling/activation. Representative Western blot results of these signalling events for each EC type are shown in Figure S2 (A–C).

**Table 2:** Signalling pathway information.

Pathways	Molecules	Molecular Weight kDa	Source	Dilution Used
MAPK	Phospho-c-jun (Ser63)	48	Rabbit	1∶1000
	Phosphor-ATF-2 (Thr71)	70	Rabbit	1∶1000
NF-kB	IκBα (L35A5)	39	Mouse	1∶1000
	Phospho-IκB-α (Ser32)	40	Rabbit	1∶1000
Rho-GTPase	Cdc42 (11A11)	21	Rabbit	1∶1000
	Phospho-Rac1/cdc42 (Ser71)	28	Rabbit	1∶1000
P13kinase/Akt	Phospho-Akt (Ser473) (D9E)	60	Rabbit	1∶2000
	Phospho-PDK1 (ser241)	68	Rabbit	1∶1000
Src	Phospho-Src (Tyr527)	60	Rabbit	1∶1000
	Phospho-Src (Tyr416)	60	Rabbit	1∶1000

## Discussion

The interactions of *P. falciparum* infected erythrocytes with various host cells, including endothelium, are thought to be involved in different features of disease, for example several reports have implicated cytoadherence of IE to EC in the cerebral microvasculature as the major process responsible for the development of human cerebral malaria [1]–[4]. There is evidence to support ICAM-1 as a receptor for *P. falciparum*-infected erythrocytes and that it plays important roles in the pathogenesis of cerebral malaria [3], [4], [9], [19], [28]. Previously, we and others have shown that co-culturing EC with IE increases ICAM-1 protein at the cell surface, and in our work this response was contact-dependent as this increase was not seen when IE and EC were co-cultured using a transwell system [28]. The present study has extended our previous work to include a range of EC types and variant parasite lines, including adhesive and non-adhesive IE, as well as a PfEMP1 null line (*Pfsbp* ko). Furthermore, we have also examined the pro-adhesiveness caused by the increase in surface expression of endothelial receptors contributed by the interaction of EC and IE during the co-culture processes. The induction of ICAM-1 on EC by IE has been variable under different conditions [9], [16], [23]–[29], [33]. However, our results clearly demonstrate that co-culture of IE with EC is able to induce the production of ICAM-1 and this level of expression is sufficient to support significantly increased IE adhesion. Indeed it is noticeable that a relatively small increase in ICAM-1 is able to support an almost equivalent level of IE binding than that seen with the much larger levels induced by TNF, which suggests that this contribution to cytoadherence is rapidly saturated. To further support our finding that the increased adhesion after co-culture was mediated by ICAM-1, we have used a blocking monoclonal antibody (mAb 15.2) against ICAM-1 [42] in the binding assays post co-culture and shown that the adhesion was reduced to background levels (Figure S4). Moreover, our data provide evidence that ICAM-1 induction is partly *Pfsbp1* dependent as with this knockout line ICAM-1 induction was lower compared to that induced by non-transgenic parasite lines in all EC types (Figure 2), and this resulted in significantly lower IE adhesion in HBMEC (Figure 3, Table 1). Skeleton-binding protein-1 (SBP-1) is localized to the Maurer's clefts where its function is to export PfEMP-1 and other parasite derived proteins to the erythrocyte membrane [43]. The knock out strain has consequently lost its ability to display PfEMP-1 on the *P falciparum*–infected erythrocyte surface resulting in loss of adhesion. This reduced induction of ICAM-1 expression by the *Pfsbp1* knockout is consistent with that seen using trypsin treatment of the co-cultured IE, which partially, but significantly, abrogated ICAM-1 induction (56%) in HUVEC (Figure 4). It is interesting to contrast the picture seen with IE with that observed with perfusion of EC with sickle erythrocytes. The latter causes a transient up-regulation of both ICAM-1 and VCAM-1 mRNA production, which leads to small but significant increases in surface expression on HUVEC of these receptors [44]. The mechanism responsible for the induction of ICAM-1 by sickle cells is not known and could be dependent upon soluble released factors as well as contact with a ‘modified’ erythrocyte. Similarly we are unable to rule of a contribution from soluble factors released by IE in the induction of cytoadherence receptors on EC, although we have demonstrated that the highest levels of adhesion are generally seen with co-culture with adherent parasite lines (ItG and A4 compared to C24 co-culture). The situation *in vivo* as to whether levels of soluble VCAM-1 and ICAM-1 are associated with severe malaria is also unclear, but generally the picture is one of a pro-inflammatory disease with concomitant increases in endothelial biomarkers in patients with severe disease [45]–[47].

The binding patterns seen with HDMEC after co-culture are complicated, for example, *Pfsbp-1* knockout IE does not increase ICAM-1 surface expression on EC but induced a similar IE binding profile to that seen with ItG co-incubation. This is probably mainly due to the presence of CD36 on HDMEC, which is constitutively expressed on this type of endothelium, but this does not fully explain the binding profiles produced by co-culture with C24 and A4 that result in lower subsequent binding than co-culture with the *Pfsbp1* knockout line. The role of CD36 in this effect is supported by the absence of this pattern in HBMEC and HUVEC, which do not express significant amounts of CD36 on their surface. Further work will be required to dissect the specific receptor contributions to adhesion in the HDMEC system.

The co-culture of IE with endothelial cells results in not only significant changes in the levels of adhesion molecules, but these translate into enhanced binding of IE to EC. Phenotypically, the effects caused by IE co-culture, particularly ItG, resemble those induced by TNF stimulation but vary according to EC type (Figure 3 & data not shown). The response of EC to several cytokines, especially TNF, has been considered as one of the important determinants of pathology during infection with a number of pathogens [48]–[50]. However, *in vitro* and *in vivo* studies do not always find a consistent correlation between TNF level and disease [51], [52], and TNF is implicated in mediating both protection and pathogenicity during malaria infection [53]. Specific MAPK pathways have been linked to severe malaria through work on the response of macrophages to *P. falciparum* GPI, normally resulting in TNF secretion. Despite this it has been difficult to find a direct link between high levels of TNF and severe malaria disease, particularly in children [54], and this could be, in part, due to the low threshold seen in our work for maximal IE adhesion at levels of receptor expression, much lower than that induced by high levels of TNF. Thus TNF may play some role, such as ensuring sufficient receptor expression for efficient cytoadherence, but that progression to severe disease such as cerebral malaria requires other factors for its aetiology. EC do not secrete appreciable amounts of TNF [55], however the interaction between ICAM-1 and IE may not only be acting as an adhesion molecule for IE, targeting them to tissues such as the brain, but also directly contributing to the inflammatory response of endothelium by altering intracellular signals [48] resulting in rearrangements of the actin cytoskeleton and activation of local inflammatory cascades (5).

In our studies on *var* gene expression by IE in different tissues from the Malawi PM study [56] we have seen tissue-specific distribution of different variants, which supports IE accumulation based on receptor usage and the potential for differential pathology in different organs due to non-random distribution of IE during sequestration. For example, cerebral endothelium performs a critical function with the blood-brain barrier (BBB) forming a tight barrier to maintain homeostasis for adjacent neuronal cells. Several lines of evidence suggest that BBB function is impaired during cerebral malaria [57]. One mechanism for this may be the intracellular signals produced by specific IE cytoadherence to EC receptors. Our previous studies have shown that JNK, ERK-1/2 and p38 MAP kinases can be activated in TNF-stimulated EC (expressing high levels of ICAM-1), which is dependent on ICAM-1 mediated IE adhesion [58]. This is consistent with the finding that cross-linking of ICAM-1 with monoclonal antibodies activates the MAPK kinases ERK-1/2 and/or JNK in EC [59], [60]. In brain microvascular EC, ICAM-1 can trigger Src tyrosine kinase activity and tyrosine phosphorylation on cortactin [61]. In the present study, we found that MAPK pathways were activated in HUVEC and HDMEC, but not in HBMEC. PI3 modulation was observed in HDMEC and HUVEC, but with HDMEC showing a significant activation in ItG co-culture, whereas PI3 was deactivated in HBMEC after co-culture with A4 and ItG. In general the intensity of signalling activation was correlated with the adhesion avidity of the parasite line, along similar lines to the behaviour observed previously [58]. Other signalling pathways are also affected by IE co-culture (Src and NFκB), again demonstrating IE and endothelial specificity for this effect, although it is possible that we have missed some events through the use of standard timings to measure a spectrum of signalling activities. Nevertheless the picture produced is a complex one, suggesting modulation of specific signalling pathways in different tissue EC in response to differential stimulation by parasite variants. Further work will be needed to dissect the critical signalling pathways contributing to the variety of EC phenotypes demonstrated on IE co-culture.

Taken together, the present study has systematically investigated the expression of adhesion receptors on EC in response to IE co-culture. It appears that increased expression of ICAM-l is responsible for at least some of the observed enhancement in IE adhesiveness. These events are mediated, in part, by surface molecules on IE, the most likely candidate being PfEMP-1, although we cannot discount a role for other surface proteins. Our findings are in line with previous data [7], [24]–[28], [62], suggesting that certain parasite variants, e.g. high avidity binders, have the ability to modulate endothelium to produce a pro-adhesive state preceding a systemic pro-inflammatory host response, and triggering specific signalling pathways that could lead to localised EC dysfunction. The induction of adhesion molecules is likely to create a vascular environment that favours adhesion events, lengthens microcirculation transient times and thus supports the concomitant recruitment of IE, platelets and (potentially) other blood cells to bind to the endothelium of microvessels, leading to the blockage of blood vessels as seen in the retina of CM patients [4], [63], [64].

In conclusion, our observations suggest mechanisms for the efficient sequestration of IE seen early in an infection in the absence of profound systemic host activation and the pathophysiology of severe malaria based on the targeted recruitment of parasite variants to specific tissues. A better understanding of these mechanisms will help us to design effective therapies aimed at alleviating the morbidity and mortality associated with severe disease.

## Materials and Methods

### Parasite culture

*Plasmodium falciparum* isolates used in this study were: ItG [37], A4 and C24 [38] and *Pfsbp-1* knockout strain (*Pfsbp1*-ko). The *Pfsbp-1* knockout line was derived from CS2, in which PfEMP-1 transport (along with other proteins) to the RBC surface is disrupted [40], [41]. Parasites were cultured *in vitro* in group O^+^ human erythrocytes in RPMI 1640 medium (supplemented with 37.5 mM HEPES, 7 mM D-glucose, 6 mM NaOH, 25 µg ml^−1^ gentamicin sulphate, 2 mM L-glutamine and 10% human serum) at a pH of 7.2 in a gas mixture of 96% nitrogen, 3% carbon dioxide and 1% oxygen. To minimize the effect of antigenic switching in culture, a batch of stabilates was prepared from post-selection cultures and used for no more than three weeks. For co-culture experiments, mature trophozoites (20–29 hours after invasion) were enriched by plasmagel flotation. For adhesion assays, trophozoites of a later stage (30–34 hours after invasion) were used. All parasite and EC cultures were regularly monitored for mycoplasma using Takara PCR mycoplasma detection kit (Cambrex Biosciences, UK).

### Trypsin treatment

Surface-exposed PfEMP1 was removed by proteolytic digestion of parasitized RBCs as described previously [28]. Briefly, IE (ItG line) were enriched by Plasmagel flotation, and washed three times with medium without sera and twice with PBS. IE were then divided into two sets, one set was kept in PBS and another set was incubated with 0.1 mg/ml TPCK trypsin (Thermo Scientific, Northumberland, UK) in PBS for 12 min at room temperature with gentle mixing several times by hand. The reaction was stopped by adding foetal calf serum (FCS) to a final concentration of 10%. Both sets were washed three times with PBS and two times with corresponding EC medium. Treated and untreated IE at 40% parasitaemia and 1% haematocrit (hct) were used in co-culture for 16 hours, after which ICAM-1 expression on the EC was measured. The use of high parasitaemia (40%) in co-culture was to mimic the *in vivo* situation in which foci of packed vessels are observed rather than uniform distribution of adherent IE within the microvasculature.

### Endothelial cells and co-culture conditions

Pooled primary human umbilical vein endothelial cells (HUVEC) and pooled human dermal microvascular endothelial cells (HDMEC) were obtained from Promocell (Heidelberg, Germany). Cells were maintained in complete growth medium supplied by Promocell according to the company's instructions. To maintain EC features and reduce inter-experiment variation, cells at passages five to six were used. The human brain microvascular endothelial cells (HBMEC) cell line was obtained through collaboration with Monique Stins (Johns Hopkins, USA) and was grown in MCDB 131 medium (Invitrogen, UK) supplemented with 10% FCS, 2 mm l-glutamine (Sigma UK), 10 ng/ml epidermal growth factor (Becton Dickinson UK) and 1 µg/ml hydrocortisone (Invitrogen UK). These cells were used up to passage 20. ECs were co-cultured with IE and uninfected RBCs as previously described [28] with modifications. Briefly, ECs were grown on 1% gelatin (Sigma, UK) coated in 25 cm^2^ flasks or 24 well plates with appropriate growth medium until confluent. Then the culture medium was replaced with either fresh growth medium (without hydrocortisone) alone or with normal erythrocytes, or with mature-trophozoite IE (C24, A4, ItG). IE were enriched by Plasmagel flotation (40% parasitaemia) and were adjusted to 1% haematocrit (hct). Fresh medium with low TNF (5 pg/ml) or high TNF (2 ng/ml) (Invitrogen, UK) were also included as controls. Co-cultures were carried out at 37°C in 5% CO2 incubator for 16 hours.

### Cytoadherence Assay

The experimental procedures for cytoadherence assays [9], [28], [65] were adapted to study the effects of co-culture on subsequent cytoadherence. ECs were seeded onto 13 mm thermanox coverslips (Nunc) coated with 1% gelatin in 24-well plates. The confluent ECs were co-cultured with normal RBC or IE of different parasite lines in complete EC medium. EC culture medium alone and TNF stimulated EC were also included as controls. After 16 hours co-culture, RBC or IE or TNF were removed and the EC washed with fresh medium once. The coverslips with co-cultured EC were picked up and dipped briefly in water to lyse adherent IEs followed by immediate neutralisation with binding buffer (RPMI 1640 medium, supplemented with HEPES and 6 mM glucose, pH 7.2) with gentle shaking to remove excess red blood cells. The washed coverslips were placed in a new 24 well plate and washed again until there was no RBC or IE on the EC visible under the microscope. IE used for adhesion assay were prepared at 1% hct and 3% parasitaemia in binding buffer. Uninfected RBCs were also cultured for at least 24 hours and used at 1% hct. 0.5 ml of IE suspension was applied to each well containing EC and incubated at 37°C for 1 h with gentle resuspension by rotation every 10 min. Unbound cells were removed by two washes in binding buffer followed by 2×30 min gravity washes. Cells were fixed with 1% glutaraldehyde/PBS for 1 h. After staining with 5% Geimsa for 30 min, coverslips were washed in water, air dried and mounted using DPX hard set mounting medium. 6–10 fields of each cover slip were counted at 300× magnification for the number of bound IE. Each experiment was performed in duplicate and on three independent occasions. Results were converted to bound IE/mm^2^.

### Flow cytometry

The expression of endothelial cell markers was measured by fluorescence-activated cell sorting (FACS) (FACScan; Becton Dickinson, San Jose, CA). Specific fluorescence-conjugated antibodies were used in this study, including: APC Mouse anti-human CD54 (ICAM-1), FITC Mouse anti-human CD36, PE Mouse anti-human CD106 (VCAM-1) and FITC Mouse anti-human CD31 (PECAM) (Becton Dickinson). Nonspecific fluorescence was assessed using corresponding isotype control antibodies. After 16 hours of co-culture, the EC culture medium including co-cultured RBC or IE was gently removed and EC washed once with fresh medium, followed by three to four further washes with phosphate-buffered saline (PBS) to remove any adherent IEs. ECs then were detached by gentle accutase (Sigma UK) treatment, washed with PBS containing 1% BSA and stained with antibodies for 60 min on ice in 100 µl of the recommended dilution. Cells were washed twice in PBS with 1% BSA and a FACS analysis was carried out using a FACS Calibur with the CellQuest Pro software (BD Biosciences). The expression of surface molecules was indicated by geometric mean of the fluorescence intensity.

### Statistical analysis

Values are reported as means ± SD for each set of experiments except for those reported for Figure 3, which are shown as means ± SEM, as the experiments were conducted independently at least 4 times. The results were evaluated with a two-tailed Student *t* test using GraphPad InStat. Statistical significance was defined as p<0.05.

## Supporting Information

Figure S1 The expression of EC adhesion receptors CD36 and CD31 after overnight co-culture with parasite lines: *Pfsbp* knockout, C24, A4 and ItG and normal RBC. The adhesion molecules on three EC types were measured by FACS. The expression level was represented as the geometric means of fluorescence intensity. Data were analysed by comparing means of each group from three independent experiments.
(TIF)

Figure S2 Endothelial cells were co-cultured with IEs for 20 min, the activation of signalling components were measured by Western blot. Shown are representatives of Western blots from the three EC types: (A) HBMEC; (B) HDMEC; and (C) HUVEC.
(TIF)

Figure S3 Eendothelial cells were grown on coverslips in corresponding growth medium without cytokine stimulation until confluent. Standard binding assays were conducted using either normal RBC or IE lines as described in the Methods section. Data shown are the mean number of IE per mm^2^ ± S.D. (n = 2).
(TIF)

Figure S4 ICAM-1 blocking antibody (15.2) inhibits binding induced by co-culture. Confluent HUVEC cells were grown on coverslips and co-cultured with ItG for 16 hours. Then the overlay cells were washed away (as described in the Methods section) and subsequently the HUVEC were analysed for their adhesion ability by static binding assays using the ItG line with or without ICAM-1 blocking antibody (mAb 15.2) or an unrelated control antibody. Data shown are the mean number of IE per mm^2^ ± S.D. (n = 2).
(TIF)

Table S1 Data (mean ± S.E.) for the co-culture IE adhesion assays described in Figure 3. The data are presented as IE bound/mm^2^.
(DOCX)
