# Supplementary figures and images for: Amplification of P. falciparum Cytoadherence through Induction of a Pro-Adhesive State in Host Endothelium
Source: PLoS One. 2011 Oct 17;6(10):e24784. doi: 10.1371/journal.pone.0024784 (PMC3197193; doi:10.1371/journal.pone.0024784)

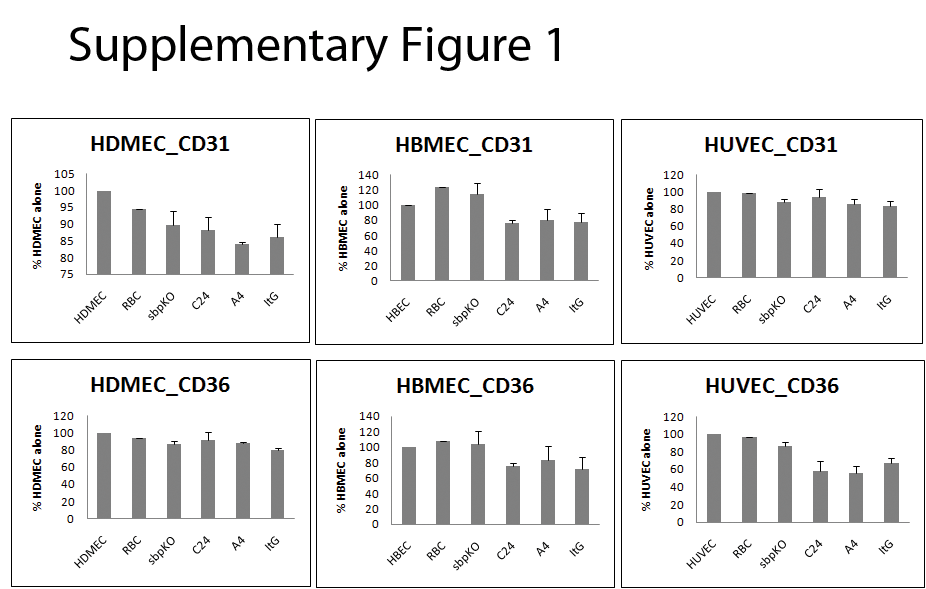

Supplement: Figure S1 — The expression of EC adhesion receptors CD36 and CD31 after overnight co-culture with parasite lines: Pfsbp knockout, C24, A4 and ItG and normal RBC. The adhesion molecules on three EC types were measured by FACS. The expression level was represented as the geometric means of fluorescence intensity. Data were analysed by comparing means of each group from three independent experiments. (TIF) [file pone.0024784.s001.tif]

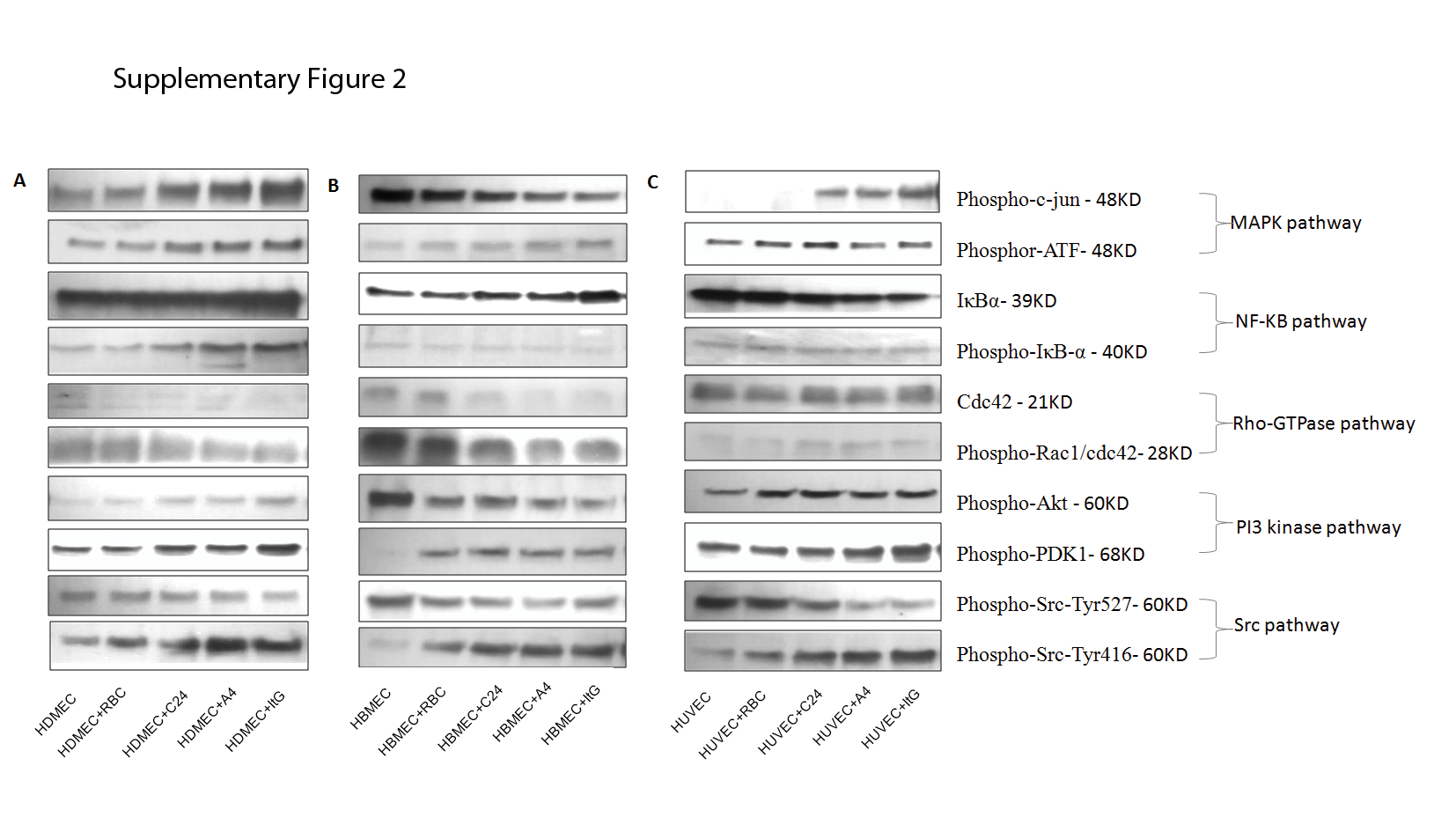

Supplement: Figure S2 — Endothelial cells were co-cultured with IEs for 20 min, the activation of signalling components were measured by Western blot. Shown are representatives of Western blots from the three EC types: (A) HBMEC; (B) HDMEC; and (C) HUVEC. (TIF) [file pone.0024784.s002.tif]

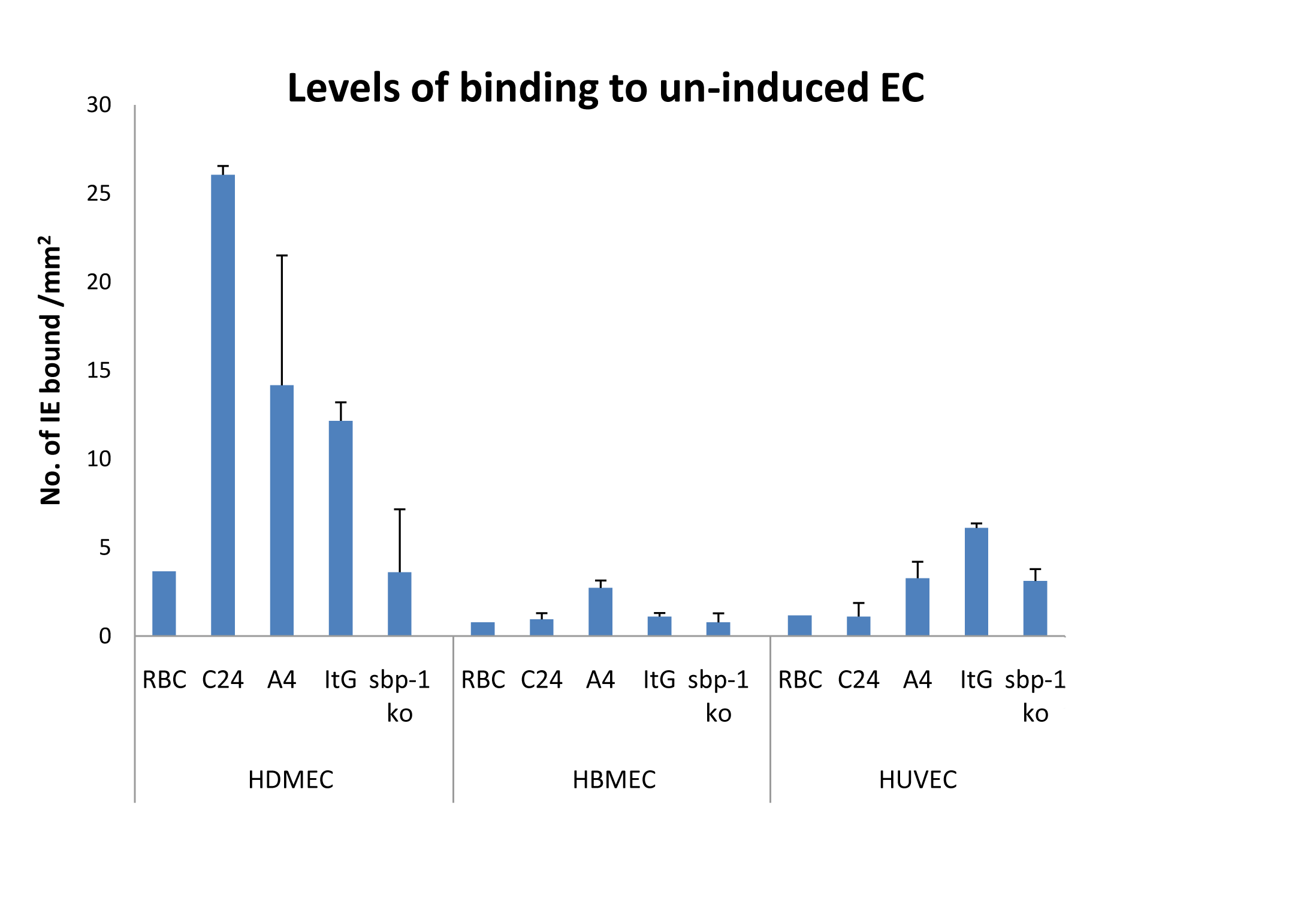

Supplement: Figure S3 — Eendothelial cells were grown on coverslips in corresponding growth medium without cytokine stimulation until confluent. Standard binding assays were conducted using either normal RBC or IE lines as described in the Methods section. Data shown are the mean number of IE per mm2 ± S.D. (n = 2). (TIF) [file pone.0024784.s003.tif]

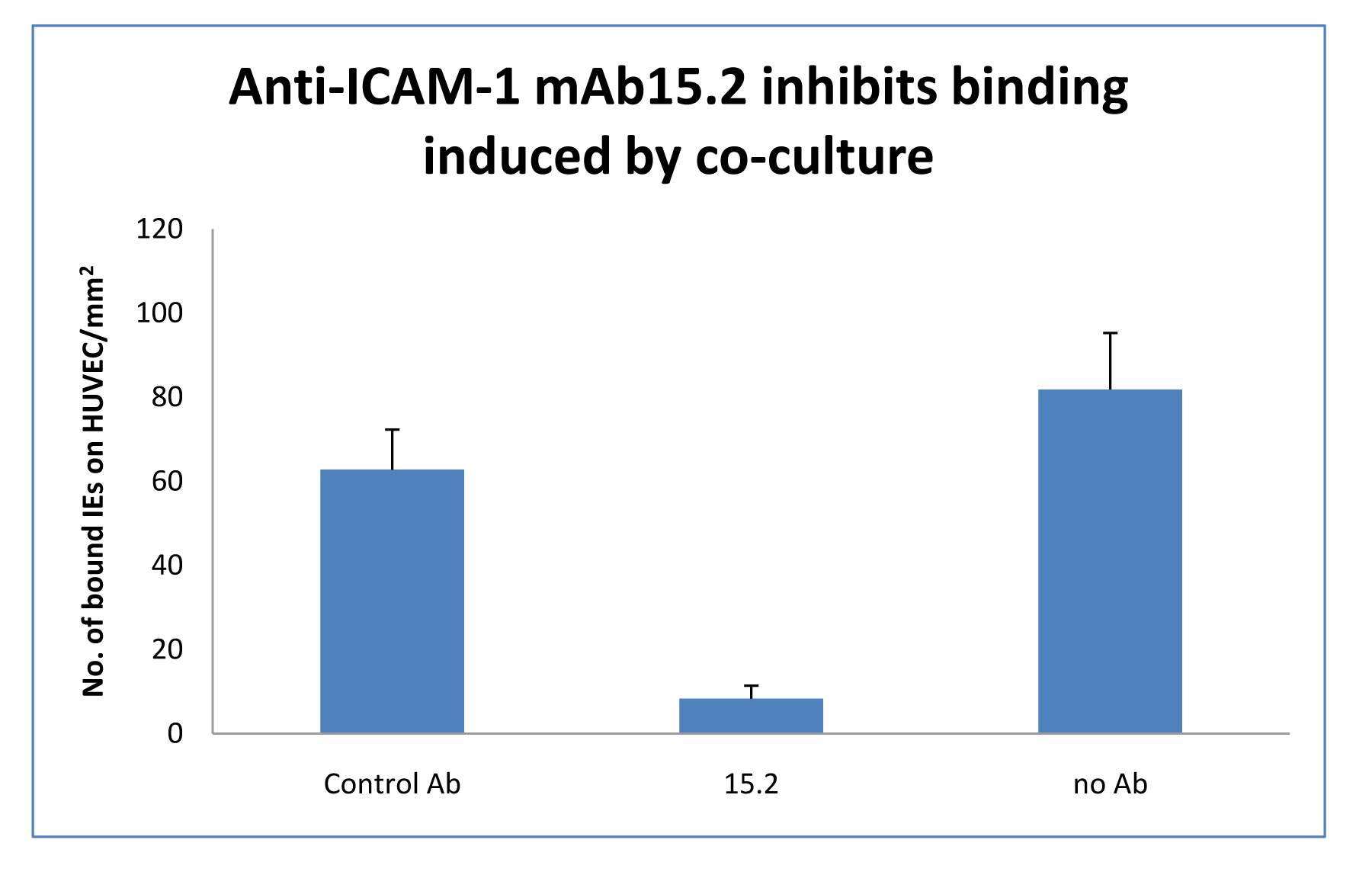

Supplement: Figure S4 — ICAM-1 blocking antibody (15.2) inhibits binding induced by co-culture. Confluent HUVEC cells were grown on coverslips and co-cultured with ItG for 16 hours. Then the overlay cells were washed away (as described in the Methods section) and subsequently the HUVEC were analysed for their adhesion ability by static binding assays using the ItG line with or without ICAM-1 blocking antibody (mAb 15.2) or an unrelated control antibody. Data shown are the mean number of IE per mm2 ± S.D. (n = 2). (TIF) [file pone.0024784.s004.tif]
