# Supplementary material for: Amplification of P. falciparum Cytoadherence through Induction of a Pro-Adhesive State in Host Endothelium
Source: PLoS One. 2011 Oct 17;6(10):e24784. doi: 10.1371/journal.pone.0024784 (PMC3197193; doi:10.1371/journal.pone.0024784)
Supplement: Table S1 — Data (mean ± S.E.) for the co-culture IE adhesion assays described in Figure 3. The data are presented as IE bound/mm2. (DOCX) [file pone.0024784.s005.docx]

Supplementary Table 1 – Data for co-culture adhesion assays (Figure 3) (IE bound/mm^2^)

|  | HDMEC | | HBMEC | | HUVEC | |
| --- | --- | --- | --- | --- | --- | --- |
| Co-culture/ Binding line | mean | SE | mean | SE | mean | SE |
| RBC/ ItG (n = 7) | 106.1 | 17.4 | 97.5 | 13.2 | 35.4 | 2.8 |
| A4/ ItG (n = 7) | 271.2 | 97.1 | 391.0 | 77.7 | 72.7 | 7.7 |
| C24/ ItG (n = 7) | 234.8 | 88.5 | 264.2 | 54.3 | 44.7 | 4.5 |
| ItG/ ItG (n = 7) | 685.3 | 27.0 | 406.0 | 79.1 | 167.5 | 72.9 |
| *Pfsbp1*/ ItG (n = 4) | 535.2 | 144.0 | 118.5 | 11.2 | 82 | 5.2 |
